# The Potential of Nrf2 Activation as a Therapeutic Target in Systemic Lupus Erythematosus

**DOI:** 10.3390/metabo12020151

**Published:** 2022-02-06

**Authors:** Michelle T. Barati, Dawn J. Caster

**Affiliations:** Division of Nephrology and Hypertension, Department of Medicine, Health Sciences Campus, University of Louisville, Louisville, KY 40202, USA; michelle.barati@louisville.edu

**Keywords:** systemic lupus erythematosus (SLE), lupus nephritis (LN), oxidative stress, Nrf2

## Abstract

Inflammation and oxidative stress are well established in systemic lupus erythematosus (SLE) and are critical to the pathogenesis of autoimmune diseases. The transcription factor NF-E2 related factor 2 (Nrf2) is a central regulator of cellular anti-oxidative responses, inflammation, and restoration of redox balance. Accumulating reports support an emerging role for the regulation of Nrf2 in SLE. These include findings on the development of lupus-like autoimmune nephritis and altered immune cell populations in mice lacking Nrf2, as well as decreased Nrf2 abundance in the dendritic cells of patients with SLE. Nrf2-inducing agents have been shown to alleviate oxidative and inflammatory stress and reduce tissue injury in SLE mouse models. Since Nrf2 expression can be increased in activated T cells, the precise role of Nrf2 activation in different immune cell types and their function remains to be defined. However, targeting Nrf2 for the treatment of diseases associated with oxidative stress and inflammation, such as SLE, is promising. As investigation of Nrf2-inducing agents in clinical trials grows, defining the signaling and molecular mechanisms of action and downstream effects in response to different Nrf2-inducing agents in specific cells, tissues, and diseases, will be critical for effective clinical use.

## 1. Introduction

Systemic lupus erythematosus (SLE) is a complex autoimmune disease with genetic, epigenetic, environmental, hormonal, and immune regulatory factors [1]. Abnormal clearance of apoptotic bodies followed by autoantibody production to nuclear antigens and loss of self-tolerance are key to SLE development. These autoantibody/antigen complexes (immune complexes) are deposited in organs and tissues causing inflammation and, ultimately, tissue damage. Both innate and adaptive immune responses contribute to the development of SLE. Neutrophils contribute through the release of neutrophil extracellular traps (NETs), which contain chromatin fibrils and histones that add to the nuclear antigen burden in patients with SLE [2]. Macrophages contribute through the decreased clearance of apoptotic bodies [3] and a shift in macrophage profiles to the more classic/M1 (pro-inflammatory) phenotype, compared to the non-classic/M2 (pro-resolving/repair) phenotype [4]. Antigen-presenting cells, especially dendritic cells, present nuclear autoantigens to T cells, leading to activation and differentiation. Nuclear antigens from apoptotic bodies and NETs also induce interferon α production by plasmacytoid dendritic cells, which amplifies immune responses. All of these cells release cytokines to continually promote inflammation, dendritic cell maturation and T cell activation [5]. Activated T cells help to induce autoreactive B cells and antibody-producing B cells. Immune complex formation further amplifies and perpetuates the immune response and inflammation. SLE T cells are further characterized by reduced interleukin 2 and increased interleukin 17 and the generation of pathogenic Th17 cells [1,6]. Kidney involvement, characterized by immune complex deposition in the kidneys, termed lupus nephritis (LN), occurs in 50–60% of patients with SLE [7]. This leads to increased morbidity and mortality in patients with SLE. Immunosuppressive regimens remain the cornerstone of LN treatments. The rate of complete response with standard of care therapies remains under 50% at 12 months [2,8]. Novel therapies having improved efficacy and reduced toxicity are needed.

An imbalance between the cellular generation of reactive oxygen species (ROS) and anti-oxidative capacity results in oxidative stress, which is exacerbated by inflammation, another key player in aging and the progression of chronic diseases such as SLE [9,10,11]. Similarly, oxidative stress can lead to, or augment, inflammation [12] and may be a key immune regulatory factor contributing to the initial aberrant SLE immune response [10]. ROS contribute to abnormal cell death signals, enhanced apoptosis, and delayed clearance of apoptotic cells [6], and oxidative modification of self-antigens can trigger autoantibody production, which can lead to organ damage in SLE. While the presence of oxidative stress in different cell and tissue types is clearly evident in SLE and exposure to increased ROS alters activation of cells (e.g., neutrophils, T cells, and peripheral blood mononuclear cells), the overall role of oxidative stress in SLE remains unclear [6,13]. For example, the failure to generate sufficient ROS for an oxidative burst in mice with a mutated neutrophil cytosolic factor 1, a subunit of NADPH oxidase, leads to development of autoimmune disease [14]. The transcription factor Nrf2 (NF-E2 related factor 2), is a central regulator of cellular anti-oxidative and anti-inflammatory responses. A growing number of reports on the regulation of oxidative and inflammatory stress support a potentially significant role for Nrf2 in SLE [6]. Nrf2 deficiency leads to lupus-like autoimmune syndrome and nephritis in aged female mice, suggesting a direct role for Nrf2 deficiency in the pathogenesis of autoimmune disease [15]. In addition, increased oxidative DNA damage, Nrf2, and an Nrf2 transcriptional target in the glomeruli of SLE patients potentially demonstrated a tissue response to oxidative stress associated with SLE [16]. Current medications used to treat LN have varying effects on Nrf2 signaling. Corticosteroids, which are widely used in SLE and LN, indirectly downregulate Nrf2 [6]. However, Nrf2 activity appears to be preserved by hydroxychloroquine and mycophenolate mofetil. Cyclophosphamide, an alkylating agent used in the treatment of LN, is associated with high oxidative stress, which can lead to hepatotoxicity and myelotoxicity, and Nrf2 activation may ameliorate these effects [6,10]. Thus, Nrf2 regulation in SLE may be critical for maintaining redox homeostasis, normal immune responses, and decreasing tissue injury.

## 2. Nrf2

**Figure 1:**
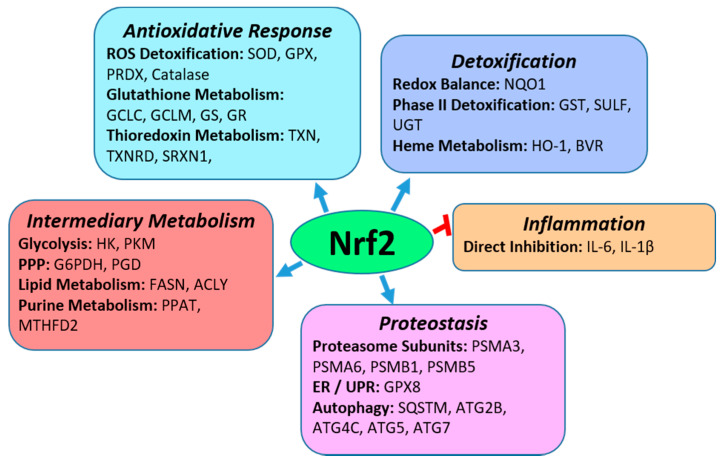
Biological Functions of Nrf2. As a transcription factor, Nrf2 is a central regulator of anti-oxidative responses and detoxification mechanisms. It also induces the expression of genes in intermediary metabolism and multiple pathways in proteostasis such as the proteasomal degradation of proteins and degradation of protein aggregates through autophagy. In addition, Nrf2 directly inhibits inflammation by inhibiting expression of IL-6 and IL-1β. Specific pathways that Nrf2 regulates in each biological functional group are in bold with examples of specific Nrf2 transcriptional gene targets in each pathway. Abbreviations of Nrf2 target genes in the pathways: SOD-superoxide dismutase; GPX, glutathione peroxidase; PRDX-peroxiredoxin; GCLC-glutamyl-cysteine ligase, catalytic; GCLM-glutamyl-cysteine ligase, modifier; GS-glutathione synthetase; GR-glutathione reductase; TXN-thioredoxin; TXNRD-thioredoxin reductase; SRXN-sulfiredoxin; NQO1-NAD(P)H dehydrogenase quinone-1; GST-glutathione-S-transferase; SULT-sulfotransferase; UGT-UDP-glucuronosyltransferase; HO-1-heme oxygenase 1; BVR-biliverdin reductase; IL-6-interleukin 6; IL-1β-interleukin 1β; PSMA-proteasome 20S subunit alpha; PSMB-proteasome 20S subunit beta; SQSTM-Sequestosome; ATG2B-autophagy related 2B; ATG4C-autophagy related 4C cysteine peptidase; ATG5 or 7-autophagy related 5 or 7; HK-hexokinase; PKM-pyruvate kinase muscular; G6PDH-glucose 6 phosphate dehydrogenase; PGD- 6-phosphogluconate dehydrogenase; FASN-fatty acid synthase; ACLY-ATP-citrate lyase; PPAT-Phosphoribosyl Pyrophosphate Amidotransferase; MTHFD2-Methylenetetrahydrofolate Dehydrogenase/Cyclohydrolase. Abbreviations in pathways (bold font): ROS-reactive oxygen species; ER-endoplasmic reticulum; UPR-unfolded protein response; PPP-pentose phosphate pathway.

Nrf2 is a central regulator of cytoprotection and redox homeostasis in cells. This is accomplished through induction of Nrf2 target genes, which include enzymes involved in antioxidative responses and phase II detoxification processes. As shown in Figure 1, Nrf2 also plays a role in inflammation, cell proteostasis through transcriptional regulation of proteasomal subunit proteins and autophagy, as well as in intermediary metabolism by regulating enzymes of the pentose phosphate pathway, purine metabolism, and lipogenesis [17,18,19,20,21].

**Figure 2:**
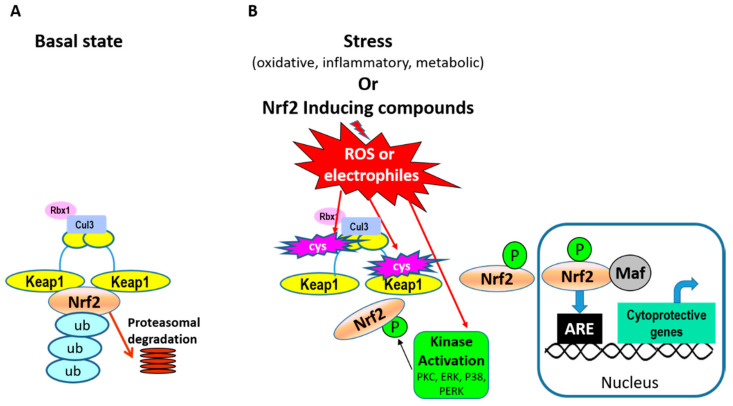
Pathways of Nrf2 activation. (**A**) In basal conditions Keap1 (Kelch ECH-associating protein 1) dimers associate with Nrf2 in the cytosol by binding to two different motifs on Nrf2. Keap1 is an adaptor protein for cullin-3 (Cul3) E3 ubiquitin ligase and its associating protein Rbx1, and this leads to ubiquitination of Nrf2 and proteasomal degradation. (**B**) In cell-stress conditions, specific cysteine residues on Keap1 are modified (marked in pink blasts), altering its association with Nrf2 and allowing Nrf2 to escape ubiquitination and proteasomal degradation. Simultaneously, stress conditions activate cell signaling pathways, kinase activation, and Nrf2 phosphorylation. Examples of kinases known to phosphorylate Nrf2 include PKC (protein kinase C), PERK (Protein kinase RNA-like endoplasmic reticulum kinase), and p38 MAPK (mitogen activated protein kinase). Together, these signals allow Nrf2 to escape proteasomal degradation and translocate to the nucleus. In the nucleus, Nrf2 forms heterodimers with small Maf proteins and binds to antioxidant-response element (ARE) sites in the promoter regions of its gene targets (examples listed in Figure 1).

Nrf2 is a “cap ‘n’ collar”-basic region leucine zipper transcription factor and its abundance is regulated through binding to Keap1 (Kelch ECH-associating protein 1) in the cytosol [22]. Binding to Keap1 is done through ETGE and DLG binding motifs on Nrf2. Keap1 is an adaptor protein for cullin-3 E3 ubiquitin ligase and this association leads to ubiquitination of Nrf2 and proteasomal degradation in unstressed cells (Figure 2A). During cell stress conditions, such as oxidative stress, Keap1 is the main sensor of stress through modification of specific cysteine residues on Keap1 by oxidants and electrophiles (Figure 2B). These modifications alter its association with Nrf2, allowing it to escape ubiquitination and proteasomal degradation and translocate to the nucleus. Stress simultaneously activates cell-signaling pathways and kinases capable of directly phosphorylating Nrf2 and modulating its localization and/or transcriptional activity. Examples of kinases known to phosphorylate Nrf2 include protein kinase C (PKC) [23], casein kinase 2 [24], mitogen activated protein kinases (MAPKs) [25], and PERK (Protein kinase RNA-like endoplasmic reticulum kinase) [26]. Direct phosphorylation of Nrf2 by PERK links it to cytoprotective signaling associated with an unfolded protein response due to endoplasmic reticulum stress. The functional consequences of Nrf2 phosphorylation at specific residues are dependent on the type of cell, stress, or stimulus. For example, phosphorylation of Nrf2 serine-40 by PKC has been shown to both increase [23] and not alter [27] Nrf2 nuclear accumulation. Similarly, regulation of MAPK signaling by pharmacologic and genetic approaches alters Nrf2 transcriptional activation [28], but substitution of specific Nrf2 residues phosphorylated by MAPKs with alanine, resulted in limited effects on Nrf2 transcriptional activation [25]. These findings suggest that phosphorylation at specific residues may not play solitary roles in Nrf2 function but rather play a part in a complex interplay of multiple signaling events and Nrf2 modifications involved in its activation. In the nucleus, Nrf2 forms heterodimers with small Maf proteins and binds to antioxidant-response element (ARE) sites in the promoter regions of its target genes to initiate transcription.

### 2.1. Nrf2 Regulation of Anti-Oxidative Responses and Cytoprotection

Nrf2 regulates redox homeostasis and combats oxidative stress through multiple mechanisms. It induces expression of enzymes directly involved in detoxifying ROS, such as superoxide dismutase (SOD), to break down superoxide, as well as glutathione peroxidases (GPXs), peroxiredoxins (PRDXs), and catalase, to metabolize hydrogen peroxide [29,30,31]. Glutathione is critical for redox homeostasis, the response to oxidative stress, and functioning of glutathione peroxidases, and its synthesis and metabolism is regulated by Nrf2. The first rate-limiting step in glutathione synthesis is the reaction of glutamate with cysteine to form γ-glutamylcysteine, which is catalyzed by glutamate cysteine ligase (GCL). GCL is composed of catalytic (GCLC) and modifier (GCLM) subunits, both of which are Nrf2 transcriptional targets [30]. Glutathione synthetase, also a target of Nrf2 [32], catalyzes the second reaction in glutathione synthesis between γ-glutamylcysteine and glycine. Nrf2 also regulates the Slc7A11 gene, which encodes the xCT subunit of the cystine/glutamate transporter. Cystine is reduced to cysteine, which shows that Nrf2 plays a role in maintaining the necessary supply of a glutathione precursor. Nrf2 also plays a crucial role in phase II detoxification processes, of which glutathione plays an important role. The breakdown/inactivation of peroxides by GPXs requires reduced glutathione, and this reaction results in the oxidation of glutathione. Oxidized glutathione is reduced by glutathione reductase (GR), another Nrf2 transcriptional target [30]. Thus, Nrf2 regulation of the glutathione precursor supply, synthesis, metabolism, and recycling, contributes to ROS detoxification. The thioredoxin (TXN) antioxidant system also contributes to the redox balance by maintaining a reducing environment. It accomplishes this by reducing disulfide bridges, and interacting with reductive enzymes like PRDXs which catalyze the reduction of hydroperoxides, hydrogen peroxide, and peroxynitrite. PRDXs are oxidized during the catalysis of these molecules, and TXN in turn catalyzes the reduction of PRXDs. Nrf2 induces expression of multiple proteins/enzymes in the TXN system, including TXN, thioredoxin reductase and sulfiredoxin [31].

Electrophilic quinones can have oxidative effects and the Nrf2 target NQO1 (NAD(P)H dehydrogenase quinone-1) is a cytoplasmic two-electron reductase [30,33] that can detoxify these species. Like quinones, heme can be a source of reactive species and heme oxygenase 1 (HO-1) is an important Nrf2-regulated cytoprotective enzyme [34] involved in oxidative heme cleavage. The reaction results in the generation of biliverdin, Fe^2+^, and carbon monoxide (CO). Biliverdin reductase, another Nrf2 target [35], reduces biliverdin to bilirubin. Bilirubin, CO, and ferritin, have antioxidant and anti-inflammatory properties [36]. Another way that Nrf2 maintains redox homeostasis is by producing NADPH, a co-factor for NQO1 and antioxidative enzymes. This is accomplished by Nrf2 induction of intermediary metabolism enzymes that generate NADPH, including glucose-6-phosphate dehydrogenase (G6PD), 6-phosphogluconate dehydrogenase (PGD), isocitrate dehydrogenase 1 (IDH1), and malic enzyme (ME) [37]. Other Nrf2-induced cytoprotective mechanisms include glutathione conjugation to endogenous reactive and xenobiotic molecules by glutathione-S-transferases for detoxification [38].

### 2.2. Nrf2 Regulation of Inflammation

Activation of Nrf2 in multiple models of a disease associated with oxidative stress and inflammation alleviates markers of injury and prevents disease progression. The majority of these effects are believed to be due to the upregulation of anti-oxidative and phase II detoxifying enzymes by Nrf2. However, it can also have a more direct role in regulation of inflammation. At the molecular level, Nrf2 inhibits transcription of pro-inflammatory *IL-6* and *IL-1β* cytokines by binding to promoter proximal regions of these genes in macrophages and inhibiting RNA Pol II recruitment [20]. In addition, Nrf2 inhibits the chemokines MIP2 (CXCL2) and MCP-1 (CCL2), because the peritoneal neutrophils in Nrf2-deficient mice showed an increased abundance of these factors in response to LPS [39]. Nrf2 also inhibits promoter activity of the adhesion molecule, VCAM1 [40], suggesting roles for Nrf2 in inhibiting adhesion and migration. There is substantial crosstalk between Nrf2 and the NFκB pro-inflammatory pathway that can play a role in regulating inflammation. Interaction of IκB kinase with Keap1 results in stabilization of the NFκB inhibitor, IκB, and thus negative regulation of NFκB [41]. In addition, MafK is a small Maf protein with which Nrf2 heterodimerizes in the nucleus. MafK promotes acetylation and the DNA-binding activity of p65 NFκB by increasing the interaction of p65 and CBP, which can be inhibited by Nrf2/MafK association [42]. Alternatively, p65 regulation of MafK/histone deacetylase 3 (HDAC3) interaction can recruit HDAC3 to Nrf2 ARE regions and suppress Nrf2 transcriptional activation [43]. Nrf2 contributes to inflammation resolution by increasing macrophage prostaglandin D synthase expression. This leads to increased producing 15d–PGJ2, 15-deoxy-12,14-prostaglandin J2, an Nrf2 inducer. Nrf2 in turn upregulates CD36 and HO-1 in macrophages to promote efferocytosis [44].

The regulation of Nrf2 can modulate immune cell responses by preventing oxidative tissue injury, apoptosis, and secondary necrosis, and thus to autoantigen production, T cell activation, and autoantibody production. The loss of Nrf2 function and detoxifying target gene induction could lead to the accumulation of reactive intermediates in tissues and the promotion of autoimmune disorders. Nrf2 may regulate T cell function by inhibiting pro-inflammatory Th1 cytokines, interferon-γ and TNFα, to promote T cell differentiation to anti-inflammatory Th2 cells [45]. Pharmacologic induction and deficiency of Nrf2 in mice is associated with altered T cell populations and macrophage polarization [46,47,48], and Nrf2 expression is increased in activated T cells [49,50,51] and non-classic/anti-inflammatory macrophages [48]. One study found that T-cell-specific Nrf2 activation increased the frequency of regulatory T cells (Tregs) and protected kidneys in a mouse model of ischemia-reperfusion injury [52]. Another report shows that systemic activation of Nrf2 in mice by Keap1 knockdown prevented inflammation and lethality in the scurfy mouse model of autoimmune disease with Treg functional deficiency [53], and this was associated with decreased activation of T cells and cytokine production. However, cell lineage-specific Nrf2 activation through targeted Keap1 disruption in T cells, myeloid cells, and dendritic cells, either had no effect or only partially alleviated inflammation in these mice. These results suggest that Nrf2 activation in multiple immune cell types is required to combat inflammation. In lipopolysaccharide (LPS)-induced sepsis, constitutive Nrf2 activation showed protection by the expansion, and metabolic re-programming, of myeloid-derived suppressor cells (MDSC) [54], thus highlighting the role of Nrf2 in immune response regulating cells. Together, these studies demonstrated a potentially significant role for Nrf2 activation in the modulation of immune cell responses and alleviation of inflammation. Whether the beneficial effects of Nrf2 induction are indirectly related to the alleviation of stress or by direct transcriptional regulation in immune cells, remains to be defined. Furthermore, the beneficial and anti-inflammatory effects of Nrf2 activation may depend on cell/tissue and disease type.

## 3. Nrf2 in SLE and LN

### 3.1. Nrf2 in Animal Models of SLE and LN

#### 3.1.1. Nrf2 Deficient Mice Develop Lupus-like Autoimmune Disease

One the most significant findings directly linking Nrf2 to SLE came from initial observations in Nrf2-deficient mice. Yoh et al. [15] first observed that *Nrf2*^−/−^ female mice over 15 months of age had shortened lifespans and were dead within 100 weeks. Female *Nrf2^−/−^* mice developed severe glomerular lesions, and histologic examination demonstrated lupus-like nephritis by lobular formation, moderate to severe cellular proliferation, segmental sclerosis, presence of crescents, and sub-epithelial electron-dense deposits. In addition to mesangial deposits, capillary deposition of IgG, IgM, and C3 were also present. Lymphocytes and macrophages made up the majority of cells in interstitial inflammation when detected. Creatinine clearance was lower in aged female *Nrf2^−/−^* mice, indicating renal function impairment. Serum IgG and anti-dsDNA, and markers for oxidative stress in subcutaneous fat tissue, were increased.

Later studies by Li et al. [55] reported similar findings in the renal pathology of 12-month-old female mice as well as increased IgG deposition in the liver, heart, and brain. Increased renal lipid peroxidation was seen in aged mice, whereas oxidative DNA damage was observed in the kidneys of *Nrf2^−/−^* female mice as young as 5 months. In addition, the kidneys, spleen, and liver of *Nrf2^−/−^* mice had higher numbers of apoptotic cells and splenocytes and exhibited higher apoptotic rates. Gene-expression changes in the liver and spleen of young mice showed downregulation of detoxification genes in male and female Nrf2-deficient mice, such as glutathione S-transferases and aldehyde dehydrogenases. Furthermore, genes involved in antigen presentation, anti-DNA autoantibody generation, development of hyperplasia in the spleen, and neutrophil and monocyte chemotaxis were increased in Nrf2-deficient female mice. These gene expression changes likely contributed to altered immune responses and disease development.

Continued investigations by Ma et al. [46] determined that *Nrf2^−/−^* mice had varying degrees of other inflammatory lesions such as lymphocytic sialitis, dermatitis, hyperkeratosis, myocarditis, vasculitis, pancreatitis, pleuritis, myocarditis with fibrosis, pericarditis, and conjunctivitis. Glomerulonephritis was the leading cause of death, and affected mice had higher blood urea nitrogen (BUN) and reduced hematocrit. Neoplasia was a cause of death in about 20% of the mice. Lymph node and spleen isolated T cells in the *Nrf2*^−/−^ mice had increased proliferation when stimulated with anti-CD3, while spleen B cell proliferation in response to LPS and anti-IgM was unchanged. Furthermore, the fraction of CD4+ lymphocytes increased while CD8+ lymphocytes decreased, suggesting dysregulated immune responses with Nrf2 loss. Lastly, this study determined that constitutive and 3-t-butyl-4-hydroxy anisol (antioxidant) inducible expression of some genes for phase II detoxification enzymes and antioxidative response were reduced in the *Nrf2*^−/−^ mice, confirming the requirement of Nrf2 for transcription of some ARE-regulated genes critical to redox homeostasis.

Table 1 compares the effects of Nrf2 deficiency in mice at various ages from studies by Yoh et al., Li et al., and Ma et al. [15,46,55], all three of which clearly showed development of an SLE-like disease. However, differences in age of disease onset or markers of tissue injury may be related to the background strain of the *Nrf2^−/−^* mice. For example, there was a difference in male and female *Nrf2^−/−^* mice survival between the studies by Yoh et al. [15] and Ma et al. [46]. In addition, kidney and liver lipid peroxidation in 24-week-old *Nrf2^−/−^* mice increased in the Ma et al. [46] study, but showed no difference in the study by Li et al. [55]. These differences may need to be considered in future study designs and data interpretation for comparative analyses. Together, these studies clearly defined a critical role for Nrf2 in preventing the development of SLE and potentially other autoimmune diseases and by highlighting its biologic effects. Furthermore, these studies support previous reports of the role of oxidative stress in dysregulated immune responses, such as lymphocyte activation from exposure to ROS [56], and the association of anti-oxidative and drug-metabolizing gene polymorphisms with SLE [57].

#### 3.1.2. Effects of Nrf2 Deficiency in Mouse Models of Spontaneous SLE-like Disease

The effects of Nrf2 deficiency were examined in MRL/*lpr* mice, which are defective in Fas-mediated apoptosis and spontaneously develope an SLE-like autoimmune disease [58] and glomerulonephritis. The Fas-mediated apoptosis defect is believed to contribute to the autoimmune disease due to the failure in induced cell death of activated T cells. Morito et al. [59] generated *Nrf2^−/−^lpr/lpr* by crossing *Nrf2^−/−^* on the ICR background strain with MRL/*lpr* mice. Surprisingly, Nrf2 deficiency prolonged the lifespan of female mice as 50% of them lived twice as long. Since renal failure is the leading cause of death in the MRL/*lpr* lupus model, the prolonged lifespan is likely due to improved renal function shown by the normalization of serum creatinine and BUN, reduced proteinuria, and improved nephritis. *Nrf2^−/−^lpr/lpr* mice had lower serum IgG and anti-dsDNA and lymphadenopathy scores. The number of apoptotic cells increased in the kidneys and spleen of *Nrf2^−/−^lpr/lpr* mice, and the survival of splenocytes from these mice in response to TNFα was reduced. Spleen-cell apoptosis and TNFα-induced splenocyte cell death were improved by administering glutathione to mice or to a culture of harvested splenocytes. The findings suggest that loss of Nrf2 enhanced apoptosis, which in turn abrogated abnormal apoptosis resulting from the *lpr* gene mutation in this lupus model. However, these effects of Nrf2 loss were not observed when *Nrf2^−/−^* mice developed on the C57Bl/6 background strain, B6.*Nrf2^−/−^*, were crossed with the B6/*lpr* mouse model of lupus [60]. The B6/*lpr* mice had the same spontaneous Fas mutation, but on the C57Bl/6 mouse background strain, and rarely developed nephritis [47]. In the crossed B6.*Nrf2^−/−^lpr/lpr* mice, early-stage nephritis increased, and this was associated with Th17 activation and an increase in Th17 relevant cytokines (Il17, Il17f, Il23, Il23r, and Rorγt), suggesting a role for Nrf2 prevention of Th17 differentiation and function in LN.

The differences in outcome with Nrf2 deficiency in these two studies were likely due to the background strain of the mice used, as disease onset and the degree of tissue lesions associated with Fas*^lpr^* mutation are strain dependent [47,61]. This mutation on the C57Bl/6 strain causes significantly lower levels of anti-dsDNA antibodies and lymphoproliferation compared to this mutation on the MRL/MpJ mice, which have much higher levels of these parameters, develope the highest amount of renal lesions, and have half the lifespan. These studies suggested that Nrf2 may play a role in resistance to disease in B6/*lpr* mice, and Nrf2 deficiency in B6.*Nrf2^−/−^lpr/lpr* mice may make these mice more susceptible to injury. Furthermore, the basal Nrf2 signaling axis and gene-target expression may be different in MRL/*lpr* and B6/*lpr* mice, and Nrf2 function may be temporally regulated in these SLE models. Thus, the specific roles of Nrf2 in different stages of the disease and the specific cell types in SLE remain to be defined.

#### 3.1.3. Nrf2 is Regulated in Experimental Models of SLE

Nrf2 expression or activity was regulated in different tissues during experimental models of SLE (Table 2). Intraperitoneal injection of mice with pristane (2,6,10,14-tetramethylpentadecane) causes SLE-like disease with autoantibodies and immune complex glomerulonephritis [62]. Glomerulonephritis typically develops months after administration of pristane. In Balb/c mice, renal Nrf2 protein abundance and expression of one of its transcriptional targets, HO-1, decreased, while NFκB expression or activation increased [63,64,65] following 6–7 months of pristane-induced lupus.

One study found no change in the expression of Nrf2 gene targets HO-1 and NQO1 [66]. MDSCs isolated from the spleen of pristane-treated mice showed decreased Nrf2 nuclear localization and accumulation in the cytosol [67]. In a separate study, transcript levels for Nrf2 and two of its transcriptional targets, and Nrf2 binding activity to consensus ARE sites, decreased in macrophages 2 weeks after pristane injection. In addition, Nrf2 expression was higher in non-classic (pro-resolving) macrophages compared to classic (pro-inflammatory) macrophages [48]. In the same study, pristane caused higher surface expression of IFN-regulated proteins Ly-6C and PDCA-1 and interferon-stimulated gene expression of *Mx1, Isg15*, and *Irf7* in the peritoneal exudate cells from *Nrf2^−/−^* mice. Combined, these studies demonstrated downregulation of the Nrf2 pathway in kidneys, MDSCs from spleen, and macrophages during pristane-induced SLE and highlighted the role of Nrf2 inhibition of interferon signaling. Alternatively, in a separate study, Jiang et al. showed increased protein abundance of Nrf2 and its target NQO1 in kidneys following 5.5 months of pristane injection [16]. The difference in renal Nrf2 protein abundance in this and former studies may be due to differences in the background strain of the mice (See Table 2). Furthermore, the study by Jiang et al. also showed pristane-induced lupus nephritis to be more severe in *Nrf2*^−/−^ mice, which were associated with increases in transforming growth factor β1, fibronectin, and iNOS.

The role of Nrf2 was evaluated in murine models of accelerated severe LN (ASLN). One ASLN model was developed with a twice-weekly intraperitoneal LPS injection into female NZB/W F1 lupus-prone mice. By five weeks, these mice show increased proteinuria and serum BUN and creatinine, and decreased Nrf2 protein abundance [68,69]. Nrf2 protein abundance also decreased in NZB/W F1 lupus-prone mice not injected with LPS [70].

Environmental toxins can cause dysregulated immune responses. The contaminant trichloroethene (TCE) and its metabolites caused autoimmune/SLE-like disease in MRL/MpJ mice [71]. This model exhibited increased anti-dsDNA antibodies and increased liver protein carbonyls indicative of protein oxidation. The livers of these mice had increased inflammation as shown by the induction of transcript and protein levels of p65 NFκB, TNFα, IL-12, and RANTES. Alternatively, Nrf2 transcript and protein abundance decreased [71]. Exposure of lupus-prone MRL/*lpr* mice to another environmental toxin, bisphenol A (BPA), increased autoantibodies, proteinuria, and caused more severe nephritis, which was attributed to aberrant autophagy and decreased Nrf2 expression [72].

These studies clearly showed that inflammation and oxidative stress in mouse models of SLE are associated with reduced Nrf2 protein abundance and transcriptional activation. Therefore, mechanisms to restore Nrf2 abundance and activity are promising for the treatment of SLE.

### 3.2. Nrf2 in Human SLE and LN

Multiple studies have shown increased levels of oxidative stress in the blood, tissues, and immune cells of SLE patients [16,73,74,75]. Furthermore, levels of oxidative stress correlate with disease activity. The Keap1/Nrf2 pathway is a vital signaling pathway for antioxidant defense; thus, Nrf2 levels typically increase in response to oxidative stress, which, if left unchecked, oxidative stress can contribute to increased apoptosis, poor clearance of apoptotic bodies, autoantibody production, and increased tissue damage [6,10]. Studies involving human SLE and LN are primarily observational, but they suggest that that Nrf2 activity is impaired, which may contribute to increased oxidative stress in SLE patients.

#### 3.2.1. Nrf2 in Human Kidneys and Whole Blood

The glomeruli of patients with LN have increased markers of oxidative stress and increased Nrf2 activation [16]. Interestingly, the level of Nrf2 did not correspond with disease severity as the glomeruli from patients with class III (focal proliferative) LN had higher levels of Nrf2 compared to those with class IV (diffuse proliferative) LN [16]. Additionally, Nrf2 expression was increased in glomeruli from other immune-mediated kidney diseases, including IgA nephropathy [16]. While Nrf2 expression was elevated in tissue, whole blood from SLE patients demonstrated lower levels of Nrf2 and SOD2 compared to healthy controls, suggesting impaired clearance of ROS [48].

#### 3.2.2. Nrf2 in Human Dendritic Cells

Plasmacytoid dendritic cells (pDCs) isolated from SLE patients with high disease activity were found to have elevated intracellular ROS compared to cells in SLE patients in remission [74]. Furthermore, Nrf2 and Keap1 levels decreased in pDCs from patients with active SLE compared to healthy controls, suggesting that regulation of oxidative stress via the Keap1/Nrf2 pathway was impaired in pDCs in these patients [74], which may contribute to the pathogenic role of pDCs in SLE.

#### 3.2.3. Nrf2 in Human T cells and NK Cells

Tandon et al. evaluated oxidative stress, Keap1, and Nrf2 in T cell and Natural Killer (NK) cell subtypes isolated from SLE patients [75]. They found a positive correlation between SLE disease activity (as assessed by SLEDAI) and ROS in helper and cytotoxic T-cells. They also demonstrated a positive correlation between disease activity and Keap1 levels in CD3^+^CD4^+^ T cells, CD3^+^CD8^+^ T cells, and CD3^-^CD56^bright^ NK cells. They found a positive correlation between disease activity and protein expression of Nrf2 in CD3^+^CD8^+^ T cells, but not with other cell types. SLE disease activity positively correlated with mRNA expression of Keap1 in CD4^+^, CD8^+^, and CD56^+^ cells and mRNA expression of Nrf2 in CD8^+^ cells [75]. The biologic effects of Keap1 and Nrf2 regulation in these cell subtypes during SLE remains to be determined.

#### 3.2.4. Nrf2 and Antioxidative Gene Polymorphisms Associated with SLE

Numerous gene polymorphisms that regulate genes involved in oxidative stress pathways have been identified in SLE: superoxide dismutase (*SOD)* [76], glutathione peroxidase (*GPx*) [76], catalase (*CAT*) [76,77], and a component of NADPH oxidase, *NCF2* [78]. Nrf2 polymorphisms are associated with LN in females with pediatric-onset SLE in a Mexican Mestizo population [79]. However, a separate study failed to show a relationship between Nrf2 polymorphisms and SLE in a Japanese population. The latter study also had a limited sample size of 51 SLE patients [80]. An integrative biology approach identified transcriptional networks, including the Nrf2 pathway, associated with CKD after evaluating 16 CKD gene loci detected in a meta-analysis of multiple GWAS studies involving 66,093 subjects [81].

## 4. Nrf2 Inducers

The promising role of Nrf2 activation to combat oxidative and inflammatory stress resulted in the investigation of a large number of Nrf- inducing compounds in vitro (using cell culture studies), in animal studies (using healthy and disease models), and in healthy human subjects or some disease states. The majority of the inducers were electrophilic and reacted with cysteine sulfhydryl groups on Keap1. Mass spectrometric and site-directed mutagenesis approaches identified specific cysteine residues on Keap1 modified in response to, and required for, activation of Nrf2 [82,83]. Three specific cysteine residues on Keap1 include Cys-151, Cys-273, and Cys-288 and are believed to be the main sensors for electrophilic inducers of Nrf2 [84]. These residues may be modified individually in combination in response to various inducers.

Nrf2 inducers can generally be divided into 5 classes based on one or more Keap1 cysteine residues with which they modify [85]). Class I electrophilic inducers prefer Cys-151 and include tert-butylhydroquinone, diethyl maleate, dimethyl fumarate (DMF), sulforaphane (SFN), nitric oxide, CDDO (2-cyano-3,12-dioxooleana-1,9(11)-dien-28-oic acid), CDDO-Im (Imidazole) and CDDO-Me (methyl ester; or bardoxolone methyl). Class II electrophilic inducers prefer Cys-288 and include 15d-PGJ2, 15-deoxy-12,14-prostaglandin J2. Class III electrophilic inducers modify Cys-151/Cys-273/Cys-288 residues and include nitrooctadec-9-enoic acid, 4-hydroxynonenal, and sodium arsenate. Class IV inducers are electrophilic and Cys-151/Cys-273/Cys-288-independent and include Nrf2 induction in response to hydrogen peroxide, prostaglandin A2, dexamethasone 21-mesylate. Keap1 cysteine residues Cys-434, Cys-266, and Cys-613 are also thought to be important for Nrf2 activation [86].

Class V Nrf2 inducers represent non-electrophilic inducers such as compounds or peptides inhibiting Keap1/Nrf2 association [87]) that effectively lead to Nrf2 induction [88,89]. In addition, endogenous protein interactions capable of disrupting Keap1/Nrf2 association and Nrf2 degradation exist, such as p21 interaction with Nrf2 [90] and p62/SQSTM association with Keap1 [91].

### 4.1. Nrf2 Inducers in Animal Models of SLE

A number of studies have investigated Nrf2-inducing compounds in mouse models of SLE. These studies highlighted the therapeutic potential of Nrf2 activation for decreasing oxidative stress and inflammation and limiting SLE progression (Table 3).

#### 4.1.1. Sulforaphane

Sulforaphane (SFN) is an isothiocyanate compound found in cruciferous vegetables such as broccoli and cabbage. It is produced from the hydrolysis of its precursor, glucoraphanin, after coming into contact and interacting with myrosinase when the plant’s structures are damaged. SFN is one the most widely studied Nrf2 inducing compounds in animal disease models and clinical studies [98]. In pristane-induced SLE, SFN decreased albuminuria and augmented renal cortical Nrf2 and NQO1 protein abundance increased by pristane [16]. In the TCE model of autoimmune/SLE-like disease, SFN decreased TCE-induced p38 and ERK MAPK phosphorylation as well as *Tnfa* and *Il12* mRNA [71].

#### 4.1.2. Dimethyl Fumarate

Dimethyl Fumarate (DMF) is a fumaric acid ester currently used as the FDA-approved drug Tecfidera to treat multiple sclerosis [99]. In pristane-induced SLE, DMF decreased glomerular injury and proteinuria [66]. Furthermore, DMF increased the renal protein levels of the Nrf2 targets HO-1 and the mRNA levels of *Nqo1*, but decreased renal MCP1 proteins, and *Mcp1*, *Il6*, *Tgfb*, and *Fn* mRNA levels. These findings showed that DMF decreased pristane-induced LN through the induction of Nrf2 and attenuation of pro-inflammatory and pro-fibrotic signaling.

#### 4.1.3. CDDO (2-Cyano-3,12-dioxooleana-1,9(11)-dien-28-oic Acid)

CDDO-Im (Imidazole) and CDDO-Me (methyl ester; or bardoxolone methyl) are synthetic oleanane triterpenoids such as oleanolic acid, synthesized in plants by the cyclization of squalene. Bardoxolone methyl has been used in preclinical and clinical studies to treat cancer [100,101] and diabetic kidney disease [102]. At nanomolar concentrations, these compounds are strong inducers of Nrf2 and promote cytoprotection, whereas at higher concentrations they activate the NFκB pathway and apoptosis [101]. In pristane-induced SLE, CDDO-Im decreased classic (pro-inflammatory) macrophages (CMs) and showed an increasing trend for pro-resolving non-classic macrophages (NCMs). This effect of CDDO-Im occurred only in B6 mice, whereas it did not alter CMs or NCMs in Balb/c mice [48]. Additionally, CDDO-Im decreased mitochondrial superoxide in CMs and NCMs of pristane-treated mice, and decreased *Ifnar1* gene expression and IFN-stimulated gene expression in macrophages.

#### 4.1.4. Epigallocatechin-3-Gallate (EGCG)

EGCG is a bioactive polyphenol from green tea and a known Nrf2 inducer [103]. Its role in lupus nephritis was examined in NZB/W F1 mice where it reduced proteinuria, serum BUN and creatinine, and nephritis without altering glomerular IgG deposition or anti-dsDNA [70]. In addition, EGCG restored Nrf2 protein expression and transcript levels of two of its gene targets, *Nqo1* and *Ho1*, while decreasing the markers of inflammasomes.

#### 4.1.5. Artemisinin Derivatives

There is a long history in traditional Chinese holistic medicine of using Artemisia annua to treat malaria [104]. Artemisinin and its derivatives Artesunate, dihydroartemisinin, and SM934, are known to induce Nrf2 [105,106,107,108] and have been used in studies of rheumatic diseases, including SLE. In pristane-induced SLE, dihydroartemisinin inhibited MDSC senescence in an Nrf2-dependent manner [67]. In the BXSB mouse strain that spontaneously developed an autoimmune/SLE-like disease, dihydroartemisinin decreased TNFα in the serum and secretion from peritoneal macrophages and decreased renal p65 subunit of NFκB [92]. In a chronic graft-vs-host disease model of lupus nephritis [109], Artemisinin decreased proteinuria and inflammatory and pro-fibrotic mediators [93]. In lupus-prone MRL/*lpr* mice, Artesunate decreased serum anti-dsDNA [94], improved renal function, and decreased renal injury and proteinuria along with renal *Il6*, *Ifn*, and *Il21* mRNA [95]. In MRL/*lpr* lupus-prone mice, another derivative, SM934, increased survival, decreased IL-6, IL-10, IL-12, and activated B cells and plasma cells [97], and lowered proteinuria, renal injury, BUN, and serum anti-dsDNA antibodies [96].

#### 4.1.6. Baicalein

Baicalein is a flavonoid from Scutellaria baicalensis Georgi roots, a traditional Chinese medicine known to combat oxidative stress partly by Nrf2 activation [64]. In pristane-induced SLE, Baicalein reduced anti-dsDNA antibodies, proteinuria, renal injury, serum cytokines (IL-1B and IL-18), and renal oxidative stress. Renal Nrf2 and HO-1 abundance increased while NFκB phosphorylation and NLRP3 were downregulated. In addition, Baicalein decreased the percentages of MDSCs in kidney, spleen, bone marrow, and peripheral blood mononuclear cells. In vitro treatment of MDSC with a specific Nrf2 inhibitor, brusatol, abolished the protective effects of Baicalein on oxidative stress and inflammation in LPS-primed MDSCs, suggesting the effects of Baicalein were Nrf2 dependent.

#### 4.1.7. Dietary Extra Virgin Olive Oil (EVOO)

Feeding mice EVOO reversed pristane-induced the SLE effects on increased serum matrix metalloproteinase 3 and decreased renal Nrf2 and HO-1 abundance. In addition, dietary EVOO attenuated pristane induction of renal p38, ERK, and JNK phosphorylation. Furthermore, EVOO decreased the production of pro-inflammatory cytokines (TNFα, IL-6, IL-10, and IL-17) in LPS-stimulated splenocytes from pristane-treated mice [65].

#### 4.1.8. Oleuropein

Pristane-treated mice fed oleuropein (an antioxidant derivative of olive leaf extract) had increased Nrf2 activation, decreased inflammatory markers, and attenuated kidney damage [63].

#### 4.1.9. Antroquinonol

Antroquinonol, an antioxidant derived from Antrodia camphorate, was explored in ASLN mice [68]. In this model, mice treated with antroquinonol beginning 2 days after the first dose of LPS, had significantly attenuated disease. Findings included decreased proteinuria, hematuria, improved kidney function, and attenuation of severe histopathologic features including cellular crescent formation, neutrophil infiltration and fibrinoid necrosis. Antroquinonol prevented ROS production and increased Nrf2 activation in the kidney.

#### 4.1.10. Citral

The effects of Citral, an anti-oxidant found in Litsea cubeba, was investigated in ASLN mice [69]. It decreased proteinuria, improved kidney function, and attenuated histopathological features including reduction in cellular crescents. Citral inhibited NLRP3 inflammasome activation, decreased ROS, and enhanced Nrf2 activation.

### 4.2. Nrf2 Inducers in Studies of Cells from SLE Patients

#### 4.2.1. Artesunate

Artesunate was explored in the regulation of macrophage migration inhibitory factor (MIF) in SLE. MIF levels were increased in serum from SLE patients and positively associated with disease activity and accumulated damage in SLE PBMCs. Artesunate inhibited IFN-inducible genes (*LY6E* and *ISG15*) in SLE PBMCs and suppressed MIF production induced by IFNα-1b stimulation [110].

#### 4.2.2. Octyl Itaconate (OI)

OI is an Nrf2 inducer that causes Keap1/Nrf2 dissociation, Nrf2 protein accumulation, nuclear translocation, and target-gene induction in THP-1 human macrophages. It also activated Nrf2 in PBMCs from SLE patients. OI inhibited mRNA expression and production of TNF-α, IL-1β, and IL-6 and NFκB activation in PBMCs, and LPS-activated THP-1 cells from SLE patients, and these effects were dependent on Nrf2 [111].

### 4.3. Potential Utilization of Nrf2 Inducers for the Treatment of SLE in the Clinical Setting

While many Nrf2-inducing compounds have been identified and investigated in preclinical studies, the use of these agents in clinical settings for therapeutic purposes has been limited. Currently, DMF (Tecfidera) has been approved by the United States Food and Drug Administration for the treatment of multiple sclerosis [99]. Similarly, Fumaderm, a drug that combines DMF with monoethyl fumarate salts, is used in Europe to treat plaque psoriasis [112]. Two studies have reported the use of fumaric acid esters for effective treatment of discoid lupus erythematosus [113,114] and severe chilblain lesions [114]. These findings, combined with the anti-inflammatory properties of DMF and its approval for treating other diseases associated with inflammation, place DMF at the forefront of autoimmune disease treatment.

Bardoxolone methyl or CDDO-Me exhibits potent antioxidative, anti-inflammatory, and anti-carcinogenic properties. Early studies in cancer patients treated with bardoxolone methyl observed decreases in serum creatinine, suggesting its use in treating chronic kidney disease (CKD). Early-phase clinical trials [115,116] and the more recent 2020 TSUBAKI [102] trial, showed promising results for the treatment of Type 2 diabetic patients with CKD. After the initial trials, clinical studies in the United States on late-stage CKD in type 2 diabetic patients were halted due to adverse events related to fluid overload and heart failure [117,118]. However, clinical trials for other forms of kidney disease such as, autosomal dominant polycystic kidney disease and Alport syndrome are currently being conducted (Clinicaltrials.gov; accessed 11/2021). A previous study identified shared transcriptional networks among 9 different forms of CKD, including lupus nephritis [81]. A network of 97 pathways were linked by shared genes and aggregated into clusters of inflammation and metabolic pathways centered on the Nrf2 pathway. Targeting Nrf2 for treatment of SLE and LN with bardoxolone methyl may be promising.

SFN is widely used in clinical studies, mostly in healthy subjects [119]. A lack of consistency in the various formulations used to examine its pharmacokinetics and pharmacodynamics may be responsible for hindering the use of SFN for therapeutic purposes. Formulations from broccoli have consisted of its precursor glucoraphanin, SFN, glucoraphanin with myrosinase, broccoli or broccoli sprouts as raw/cooked/dried preparations, or extracts of broccoli seeds or sprouts.

Limitations to use of the three electrophilic Nrf2 inducers discussed thus far may be due to the non-specificity from modification of cysteine residues on proteins other than Keap1, leading to unintended responses or adverse events. Alternatively, complementary signaling events may be triggered to augment intended cytoprotective and therapeutic effects. Approaches with higher specificity may result from peptides or chemicals that disrupt Keap1/Nrf2 protein–protein interactions [87,88].

In addition, limitations to understanding downstream signaling and the potentially divergent effects of different Nrf2 inducers may hinder therapeutic uses in clinical settings. For example, studies in a mouse model of lung cancer by To et al., showed opposing effects on the size and number of tumors and differential induction of Nrf2 transcriptional gene targets in response to bardoxolone methyl, CDDO-Im, and DMF [120]. A recent review by Yagishita et al. [119] of Nrf2 biomarkers in clinical trials highlighted bardoxolone methyl, DMF, and SFN as Nrf2 inducers known to target Nrf2 in pre-clinical studies. Of these three compounds, SFN is the most widely studied compound with more than 75 published reports from clinical studies. Furthermore, compared to DMF and bardoxolone methyl, reports with SFN include more mechanism-based biomarkers measured in categories such as Nrf2 target genes, gene expression/function, inflammation, oxidative stress, carcinogen metabolites, and metabolomics. When comparing these categories among SFN, DMF, and bardoxolone methyl, studies examining Nrf2 target genes comprised around 20, 2.5, and 8% of the total publications for each inducer, respectively. Markers of inflammation were examined in 24, 7.5, and 16% of publications for SFN, DMF, and bardoxolone methyl, respectively, and for oxidative stress markers, 15, 0, and 0%. Therefore, as previously shown by To et al. [120] defining cellular targets and downstream effects for different Nrf2 inducers in specific disease states, tissues, and cell types, is critical for achieving effective therapy without off-target or adverse effects in a clinical setting.

## 5. Conclusions

A critical role for Nrf2 in preventing SLE and autoimmune disorders by regulating redox homeostasis, inflammation, and immune responses, is apparent from numerous pre-clinical studies. These include development of an SLE-like autoimmune disorder and inflammation in Nrf2-deficient mice, downregulation of Nrf2 protein abundance and target gene expression in animal models of SLE, and the effects of Nrf2-inducing agents to combat oxidative and inflammation markers and SLE pathogenesis. These studies support previous reports on the role of oxidative stress in dysregulated immune responses and highlight the potential significance of antioxidative and drug metabolizing gene polymorphisms associated with SLE. Whether the beneficial effects of Nrf2 induction are indirectly related to the alleviation of stress or to the direct transcriptional regulation in immune cells remains to be defined. Furthermore, the beneficial and anti-inflammatory effects of Nrf2 activation may depend on the type of cell/tissue type and inflammation-associated disease. Targeting Nrf2 for therapeutic purposes is likely to be promising for the treatment of SLE. Defining cellular targets and downstream effects in response to different Nrf2-inducing agents in specific disease states, tissues, and cell types, is critical for the effective use of these compounds in a clinical setting.

## Figures and Tables

**Table 1:** Biological effects in *Nrf2^−/−^* mice.

*Nrf2^−/−^*				
Strain	Age (Weeks)	Sex	Effects in *Nrf2^−/−^* Mice	Refs.
ICR	25	M and F	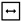 kidney glomerular lesions	Yoh et al. [15]
	25	M and F	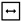 serum IgG, anti dsDNA	
	25	M and F	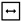 creatinine clearance	
	50	F	↑ spleen/body weight ratio;	
			germinal center hyperplasia	
	60	F	↑serum IgG, anti dsDNA	
	60	F	↑ kidney glomerular lesions	
	60	F	↓ creatinine clearance	
	60	F	↑ lipid peroxidation in subcutaneous fat	
	60	F	↓ CD19-CD3+ and CD4+CD8- lymphocytes	
	100	F	none survived	
	15; 25; 50; 70; 100	F	survival rate (%): 100; 100; 75–80; 60; 0	
	15; 25; 50; 70; 100	M	survival rate (%): 100; 80; 65; 60; 20–25	
C57B6/129SVJ	20	F	some kidney glomerular IgG, IgM, C3	Li et al. [55]
			deposition	
	20	F	↑ liver and kidney oxidative DNA damage	
	20	M and F	↓ expression of detoxification genes in	
			liver and spleen	
	24	M and F	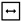 kidney and liver lipid peroxidation	
	24	M and F	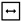 anti ds-DNA	
C57B6/129SVJ	48	F	↑anti ds-DNA	Li et al. [55]
	48	F	substantial renal glomerular IgG, IgM, C3	
			deposition	
	48	F	liver IgG, IgM deposition	
	48	F	heart, brain IgG, IgM, C3 deposition	
	48	F	↑ kidney lipid peroxidation	
	48	M and F	↑ liver lipid peroxidation	
	48	F	↑ liver and kidney oxidative DNA damage	
	48	F	↑ kidney, liver, and spleen cell apoptosis	
	36–48	F	↑ spontaneous apoptotic rate in	
			splenocytes	
129SVJ	24	F	↑ kidney and liver lipid peroxidation	Ma et al. [46]
	36	not	average age for development of	
		specified	glomerular lesions	
	48	F	↑ kidney and liver lipid peroxidation	
	15; 25; 50; 70	survival rate (%): 71; 50; 40–45; 20–25	F	
	15; 25; 50; 70	M	survival rate (%): 95; 85; 70; 55	

Abbreviations: M, male; F, female. ↓, decreased; ↑, increased, 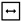, not changed or, not different.

**Table 2:** Regulation of Nrf2 in Animal Models of SLE.

*Model of SLE*				
Duration		Effect on		
(Mouse Strain)	Tissue/Cells	Nrf2/Nrf2 Targets	Other Pathways	Refs.
***Pristane***				
1–2 weeks	peritoneal	*↓Nrf2, Gpx4, Prdx1*	↓HSP70 protein	Han et al. [48]
(C57BL/6)	exudate	*Gclc, Nqo1, Sod2,*	↑mitochondrial superoxide	
	macrophages	*Gsr, Srxn* gene		
		expression		
		↓Nrf2 binding to		
		ARE-motif		
5 months	kidney	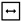 HO-1 protein	↑MCP1 protein	Ebihara et al. [66]
(Balb/c)		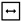 *Nqo1* mRNA		
5.5 months	Kidney	↑Nrf2, NQO1 protein	↑*iNos, Tgfb1, Nqo1, Fn* mRNA	Jiang et al. [16]
7 months	kidney	↑Nrf2, NQO1 protein	↑*iNos, Fn* mRNA; ↑p-NFκB-p65	
(C57B/SV129)				
6 months	Kidney	↓Nrf2, HO-1 protein	↑NFκB-p65, p-STAT3	Castejon et al. [63]
(Balb/c)			↑p-p38, p-JNK, p-ERK	
			↑iNOS; ↑mPGES-1, PGE2	
			↑NLRP3, IL-1β, IL-18	
6 months	Kidney	↓Nrf2, HO-1 protein	↓IκBα; ↑p-STAT3	Aparicio-
(Balb/c)			↑mPGES-1, PGE2	Soto et al. [65]
			↑p-p38, p-JNK, p-ERK	
7 months	Kidney	↓nuclear Nrf2	↑p-NFκB;	Li et al. [64]
(Balb/c)		↓HO-1 protein	↑NLRP3, cleaved casp1, IL-1β	
			↑ROS, GPx activity	
7 months	MDSC	↓nuclear Nrf2	↑*Il1b, Il6, Il8, Tnfa* mRNA	Li et al. [67]
(Balb/c)	(spleen)	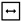 HO-1 protein	↑senescence markers (p21,	
			p53, p21)	
			↑iNOS, p47phox	
***ASLN***				
5 weeks	kidney	↓nuclear Nrf2	↑IL-6, p47phox; ↑GPx activity	Tsai et al. [68]
(NZB/NZW)				
5 weeks	kidney	↓nuclear Nrf2	↑NLRP3, IL-1β	Ka et al. [69]
(NZB/NZW)			↑p47phox, COX-2, PGE2	
***NZB/W F1***				
8.5 months	kidney	↓nuclear Nrf2	↑p47phox; ↑GPx activity	Tsai et al. [70]
		↓*Ho1, Nqo1* mRNA	↑NLRP3, IL-1β, cleaved casp1	
***BPA exposure***				
6 weeks	kidney	↓Nrf2 protein	↑NFκB-p65; ↓mTOR	Dong et al. [71]
(MRL/*lpr*)			Abnormal autophagy signaling	
			↑ERα and AhR expression	
***TCE-induced***				
6 months	liver	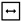 Nrf2 protein	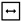 NFκB, iNOS	Banerjee et al. [72]
			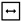 p-p38, p-JNK, p-ERK	
9 months		↓Nrf2 protein	↑NFκB, iNOS	
			↑p-p38, p-JNK, p-ERK	
			↑IL-12 protein and mRNA	
			↑protein carbonyls	
13 months		↓Nrf2 protein	↑NFκB, iNOS	
(MRL/MpJ)			↑p-p38, p-JNK, p-ERK	
			↑IL-12, TNFα, RANTES protein	
			↑*Il12, Rantes* mRNA	
			↑protein carbonyls	

Abbreviations: Gpx, glutathione peroxidase; Prdx, peroxiredoxin; ARE, Antioxidant response element; HO-1, Heme Oxygenase-1; NQO1, NAD(P)H Quinone Dehydrogenase 1; sod-superoxide dismutase GCLC-Glutamate-Cysteine Ligase Catalytic Subunit; Gsr-Glutathione Disulfide Reductase; srxn-Sulfiredoxin; HSP70-heat shock protein70; MCP1-monocyte chemoattractant protein-1; iNOS- inducible nitric oxide synthase; TGFβ-transforming growth factor β; FN-fibronectin STAT3-signal transducer and activator 3; p-p38-phospho p38 mitogen activated kinase (MAPK); p-JNK-phospho c-Jun N-terminal kinase; p-ERK-phospho extracellular signal-regulated kinase; mPGES1-Microsomal prostaglandin E synthase-1; PGE2-prostaglandin E2; IL-1-interleukin 1; NLRP3-NOD-, LRR- and pyrin domain-containing protein 3; casp-caspase; ROS-reactive oxygen species; TNF-tumor necrosis factor; COX-2-cyclooxygenase 2; ERα-estrogen receptor α; AhR-arylhydrocarbon receptor; RANTES-Regulated on Activation, Normal T Cell Expressed and Secreted; NFκB-Nuclear Factor kappa-light-chain-enhancer of activated B cells; IκB-inhibitor of nuclear factor kappa B. ↓, decreased; ↑, increased, 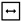, not changed or, not different.

**Table 3:** Effect of Nrf2 Inducers in Animal Models of SLE.

Nrf2 Inducer	Model of SLE	Effects	Ref
Sulforaphane	TCE-induced	↓ p38 and ERK MAPK phosphorylation	Banerjee et al. [71]
		↓ *Tnfa* and *Il12* mRNA	
	Pristane-induced	↓ albuminuria	Jiang et al. [16]
		Augmented renal Nrf2 and NQO1 protein abundance	
Dimethyl	Pristane-induced	↓ glomerular injury and proteinuria	Ebihara et al. [66]
Fumarate		↑ HO-1 protein, *Nqo1* mRNA	
		↓ MCP1 protein and mRNA; *Tgfb* and *Fn* mRNA	
CDDO-Im	Pristane-induced	↓ classic macrophages in B6 mice	Han et al. [48]
		↓ mitochondrial superoxide in macrophages	
		↓ macrophage *Ifnar1* and IFN-stimulated gene expression	
Baicalein	Pristane-induced	↓ anti-dsDNA antibodies, proteinuria, renal injury	Li et al. [64]
		↓ serum IL-1B and IL-18, and renal oxidative stress	
		↑ Renal Nrf2 and HO-1 and phospho-NFκB and NLRP3	
		↓MDSCs in kidney, spleen, bone marrow, and PBMCs	
Extra virgin olive oil	Pristane-induced	restored serum MMP3, renal Nrf2 and HO-1 abundance	Aparicio-Soto
		attenuated renal p38, ERK, and JNK phosphorylation	et al. [65]
		↓ LPS-induced TNFα, IL-6, IL-10, and IL-17 in splenocytes	
Oleuropein	Pristane-induced	↓ inflammatory markers and renal injury, Nrf2	Castejon et al. [63]
Dihydro-	Pristane-induced	inhibit MDSC senescence	Li et al. [67]
artemisinin			
	BXSB mice	↓ serum and macrophage secretion of TNFα; renal NFκB	Li et al. [92]
Artemisinin	chronic graft	↓ proteinuria; ↓ inflammatory, pro-fibrotic mediators	Wu et al. [93]
	vs. host disease		
			
Artesunate	MRL/*lpr*	↓ anti-dsDNA; ↓ proteinuria; improved kidney function	Jin et al. [94]
		↓ renal *Il6*, *Ifn*, and *Il21* mRNA	Dang et al. [95]
SM934	MRL/*lpr*	↓ anti-dsDNA; ↓ renal injury, proteinuria, serum BUN	Hou et al. [96]
		↓ IL-6, Il-10, Il-12, activated B cells and plasma cells	Wu et al. [97]
Antroquinonol	ASLN mice	↓ proteinuria, hematuria, kidney injury	Tsai et al. [68]
		improve kidney function	
		↑ Nrf2 activity and ↓ ROS in kidney	
Citral	ASLN mice	↓ proteinuria, renal injury; improved kidney function	Ka et al. [69]
		↑ Nrf2 activity; ↓ ROS and NLRP3 inflammasome	
EGCG	NZB/W F1 mice	↓proteinuria, serum BUN and creatinine, and nephritis	Tsai et al. [70]
		unaltered glomerular IgG deposition or anti-dsDNA	
		restored Nrf2 protein, *Nqo1* and *Ho1* mRNA	
		↓ inflammasome markers	

Abbreviations: EGCG-Epigallocatechin-3-gallate; p38-p38 mitogen activated protein kinase; TNF-tumor necrosis factor; IL-12-interleukin 12; HO-1, Heme Oxygenase-1; NQO1, NAD(P)H Dehydrogenase Quinone 1; MCP1-monocyte chemoattractant protein-1; TGFβ-transforming growth factor β; FN-fibronectin; IFN-interferon; NFκB-Nuclear Factor kappa-light-chain-enhancer of activated B cells; NLRP3-NOD, LRR and pyrin domain-containing protein 3; MDSC-Myeloid-derived suppressor cells; PBMC-peripheral blood mononuclear cell; JNK-c-Jun N-terminal kinase; LPS-lipopolysaccharide; ROS-reactive oxygen species; BUN-Blood Urea Nitrogen; TCE-trichloroethene; ASLN-accelerated severe lupus nephritis; ERK-extracellular signal-regulated kinase. ↓, decreased; ↑, increased.
